# From Extra Virgin Olive Oil to Refined Products: Intensity and Balance Shifts of the Volatile Compounds versus Odor

**DOI:** 10.3390/molecules25112469

**Published:** 2020-05-26

**Authors:** Jing Yan, Martin Alewijn, Saskia M. van Ruth

**Affiliations:** 1 Food Quality and Design Group, Wageningen University and Research, 6700AA Wageningen, The Netherlands; jing.yan@wur.nl; 2 Wageningen Food Safety Research, Wageningen University and Research, 6700AE Wageningen, The Netherlands; martin.alewijn@wur.nl

**Keywords:** extra virgin olive oil, odor quality, processing grades, quantitation, VOCs proportion

## Abstract

To explore relationships between the volatile organic compounds (VOCs) of different grades of olive oils (OOs) (extra virgin olive oil (EVOO), refined olive oil (ROO), and pomace olive oil (POO)) and odor quality, VOCs were measured in the headspace of the oils by proton transfer reaction quadrupole ion guide time-of-flight mass spectrometry. The concentrations of most VOCs differed significantly between the grades (EVOO > ROO > POO), whereas the abundance of *m/z* 47.012 (formic acid), *m/z* 49.016 (fragments), *m/z* 49.027 (fragments), and *m/z* 115.111 (heptanal/heptanone) increased in that order. Although the refined oils had considerably lower VOC abundance, the extent of the decline varied with the VOCs. This results in differences in VOCs proportions. The high VOC abundance in the EVOO headspace in comparison to ROO and POO results in a richer and more complex odor. The identified C5–C6 compounds are expected to contribute mainly to the green odor notes, while the identified C1–C4 and C7–C15 are mainly responsible for odor defects of OOs. Current results reveal that processing strongly affects both the quantitative and relative abundance of the VOCs and, therefore, the odor quality of the various grades of OOs.

## 1. Introduction

Olive oils (OOs) are very popular with customers due to their pleasant flavor and odor, as well as their health benefits. The concentrations of the volatile compounds (odor profile) in OOs are affected by many factors, including the cultivar [1], environmental factors [2], olive fruit maturity [3], the technical processing [4], as well as storage of the fruit (long time storage may be responsible for odor defects) [5], or storage of OOs (oxidative degradation) [6]. Therefore, OOs come with great variation in odor quality. Among those factors, processing methods (e.g., cold pressing and refining steps) cause dramatic effects on the concentrations of the volatile organic compounds (VOCs) in different grades of OOs [7], such as extra virgin olive oil (EVOO), refined olive oil (ROO), and pomace olive oil (POO).

The VOC molecules comprising 5 and 6 carbon atoms (called C5 and C6 compounds), which are mainly produced through enzymatic reactions leading to degradation of polyunsaturated fatty acids during processing [8], are considered the most important VOCs for the green odor notes of EVOO [9,10]. These compounds are more likely to be formed under cold-pressed conditions. Some oils are subjected to refining and most of C5 and C6, as well as many other VOCs are removed during the deodorization step in the refining process [11,12]. VOCs of OOs have been studied before [13,14,15], but so far, the differences in headspace concentrations of the VOCs of different OO grades has not yet been studied in depth. Therefore, it may be interesting to understand the relationship between the variation of the VOCs concentrations and the processing methods, as well as the relationship between the variation of the VOCs concentrations and the odor quality of the oils.

Proton transfer reaction time-of-flight mass spectrometry (PTR-ToF-MS) is based on soft chemical ionization by proton transfer from hydronium ions [16,17]. It is free of complex sample pre-processing and can provide VOCs fingerprints within seconds. Thus, it has been considered as an alternative method for the real-time and rapid analysis of OOs. PTR-ToF-MS has been used to study for example olive fruits [18], coffee [19], honey [20], peppers [17], ham [21], milk [16,22], and chocolate [23]. Although PTR-ToF-MS has been carried out for analyzing the VOCs of OOs [7,24,25] too, the influence of the OO grades on VOCs has not been compared extensively so far. Taiti and Marone [7] investigated the capability of PTR-ToF-MS in grading OOs, but the effect of the processing on the quantitative and relative abundance of the VOCs was not considered. Furthermore, PTR-ToF-MS coupled with a quadrupole ion guide (PTR-QiToF-MS), which has an improved transmission efficiency of ions and thus an increased sensitivity of the ion detection, has not been applied to measure the VOCs in OOs of different processing grades.

The present work is designed to elucidate the quantitative and relative differences of the VOCs in different grades of OOs (EVOO, ROO, and POO) by PTR-QiToF-MS and to explore the odor quality of the corresponding oils.

## 2. Results and Discussion

### 2.1. PTR-QiToF-MS Spectral Profile

**Figure 1:**
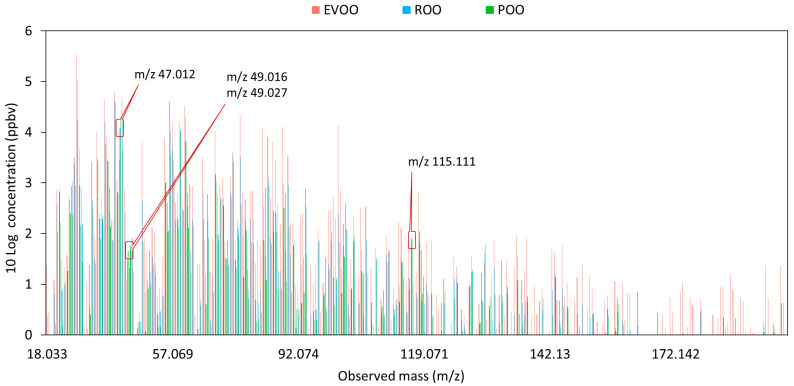
^10^Log averaged spectral profiles of volatile organic compounds of three olive oil grades (extra virgin olive oil, EVOO; refined olive oil, ROO; pomace olive oil, POO). The four highlighted mass peaks (*m/z* 47.012, *m/z* 49.016, *m/z* 49.027, and *m/z* 115.111) were present in significantly higher abundance in the headspace of ROO and POO compared to EVOO.

Two hundred OOs were subjected to PTR-QiToF-MS analysis, and 295 mass peaks in the range of *m/z* 18.033 to *m/z* 207.204 were obtained for each sample. The ^10^log transformed mass spectra for three grades, i.e., EVOO (*n* = 140), ROO (*n* = 45), and POO (*n* = 15), are presented in Figure 1. For each average spectral profile in Figure 1, the summed observed concentration of the mass peaks ranging from *m/z* 18.033 to *m/z* 207.204 in the headspace of the samples were calculated. The value for EVOO (782 parts per million by volume (ppmv)) was about 3 times higher than the summed value for ROO (276 ppmv) and 5 times higher than that value for POO (127 ppmv). This indicates that EVOO is richer in VOCs than the other lower grades of OOs. Furthermore, the concentration of most mass peaks measured in the headspace of the EVOO samples is considerably higher than the other two grades. It further supports the idea that large amounts of the VOCs are removed during the refining process [11,26].

### 2.2. Concentration Differences of the VOCs

To explore the differences between EVOO and the lower grades of OOs, the 295 mass peaks were subjected to non-parametric Kruskal–Wallis tests (*p* < 0.05). Subsequently, 291 out of 295 mass peaks were present in significant differences across the three types of OO. Fifty-four mass peaks were tentatively identified based on their accurate molar masses and likely chemical formulas [16], as well as based on information from literature [9]. The headspace concentrations, odor characteristics, and odor thresholds (OTs) of the 54 tentatively identified VOCs of the three types of OO (EVOO, ROO, and POO) are listed in Table 1. The carbon numbers of the 54 tentatively identified VOCs varied from 1 to 15 (C1–C15). Furthermore, 20 out of 54 mass peaks were tentatively identified as several possible isomeric compounds. 

#### 2.2.1. VOCs with Higher Concentrations in the EVOO Headspace 

Among the 295 mass peaks, 287 mass peaks were present in significantly higher concentrations in the headspace of the EVOO samples than for the other OOs. It confirms that most of the VOCs are removed during the refining process [11]. Similarly, for 51 out of the 54 tentatively identified VOCs, significantly higher concentrations were observed for EVOO than for the other oils (Table 1).

Except for the mass peak *m/z* 47.012 (formic acid), significantly higher headspace concentrations were determined for all identified C1–C4 compounds in EVOO compared to ROO/POO. Mass peak *m/z* 33.033 (methanol) was the most abundant compound in the EVOO headspace, followed by mass peaks *m/z* 45.034 (acetaldehyde), *m/z* 47.049 (ethanol), *m/z* 43.018 (esters), *m/z* 57.033 (2-propenal), and *m/z* 61.028 (acetic acid). However, the detection of methanol is rarely reported, and/or methanol is often found to be present at low concentrations using gas chromatography-mass spectroscopy-based techniques. There are several possible reasons for the detection of high concentrations of the VOCs with low molecular mass in the headspace of OOs using PTR-QiToF-MS. One of the possible reasons is that this mass may be a mixture of a small amount of methanol and a large amount of fragments from higher molecular masses. It may also be due to differences in the set-up between the current method and gas chromatography approaches. For instance, the way of sampling and injection differ considerably.

Significantly higher concentrations were determined for all identified C5 and C6 compounds in the EVOO headspace compared to the lower grades of OOs. Regarding C5 compounds, the headspace concentration in EVOO of mass peak *m/z* 87.081 (13 ± 8 ppmv, 3-penten-2-ol/2-methyl-3-buten-2-ol/2-methylbutanal/3-methylbutanal/pentanal/3-pentanone) was higher than *m/z* 85.064 (7 ± 6 ppmv, trans-2-pentenal/1-penten-3-one/trans-2-methyl-2-butenal) and *m/z* 103.075 (208 ± 98 parts per billion by volume (ppbv), ethyl propionate/3-methylbutanoic acid/pentanoic acid). This may be due to the malaxation step, which involves temperatures that favor amino acid conversion. This conversion results in an elevated production of 2-methylbutanal and 3-methylbutanal [8]. Among the eight identified C6 compounds, mass peak *m/z* 99.081 (13 ± 11 ppmv, trans-2-hexenal/cis-3-hexenal) were present in highest abundance in the EVOO headspace. Two isomers were tentatively identified in mass peak *m/z* 99.081, which are trans-2-hexenal and cis-3-hexenal.

Furthermore, significantly higher concentrations of 28 out of 30 identified C7–C15 compounds were determined in the headspace of EVOO than for the other OO counterparts. Among these 28 identified compounds, the concentrations of mass peaks *m/z* 93.070 (254 ± 223 ppbv, toluene, C1-benzene), *m/z* 105.09 (324 ± 247 ppbv, ethenyl benzene, C2-benzene) and *m/z* 107.086 (338 ± 275 ppbv, ethyl benzene, C2-benzene) found in the headspace of EVOO were higher than the other compounds. It is reported that oils can be easily contaminated by these potentially harmful VOCs because of their lipophilic nature and widely distribution [45]. The presence of these compounds in OOs is likely due to the contamination by gasoline vapors in the oil mill [46,47]. These compounds could also originate from the packaging materials [48]. The contamination of these compounds in OOs deserves a special consideration in the future due to their potential harm to human body [49]. Furthermore, significantly higher concentrations of those three compounds in the EVOO headspace compared to the other OOs is likely due to removal during the refining process [48].

#### 2.2.2. VOCs with Higher Concentrations in the ROO/POO Headspace

It is interesting to note that four mass peaks (*m/z* 47.012, *m/z* 49.012, *m/z* 49.027, and *m/z* 115.111) were present in significantly higher headspace concentrations in ROO and POO compared to EVOO (Figure 1 and Table 1).

Mass peak *m/z* 47.012 was tentatively identified as formic acid [31,32]. The headspace concentration of formic acid in EVOO (3 ± 3 ppmv) was significantly lower than in ROO (12 ± 11 ppmv) and POO (17 ± 16 ppmv). One of the possible pathways contributing to the formation of formic acid (*m/z* 47.012) in the lower grades of OOs is oxidation during storage [31], such as the decomposition of unstable volatiles (2,4-(E-E)-decadienal) [31,50]. Another possible pathway is microbial metabolism during storage [51]. The other two mass peaks *m/z* 49.012 and *m/z* 49.027 (non-identified) were also present in significantly lower concentrations for EVOO compared to ROO and POO. They are most likely fragments of higher molecular masses. In addition, the concentration of mass peak *m/z* 115.111 (51 ± 31 ppbv, heptanal/heptan-2-one) for EVOO was significantly lower than that for ROO (81 ± 65 ppbv) and POO (77 ± 63 ppbv). This mass peak was tentatively identified as heptanal or heptan-2-one (Table 1) [9,39], which originates from the decomposition of linoleic acid [43]. The formation of this compound in ROO and POO most likely occurs during storage, because the steam deodorization step before storage would have removed such organic compounds [12,52]. 

Taken together, the concentrations of most VOCs were significantly lower in the headspace of OOs that have been subjected to a refining step, whereas the concentrations of four mass peaks, i.e. *m/z* 47.012 (formic acid), *m/z* 49.016 (non-identified), *m/z* 49.027 (non-identified) and *m/z* 115.111 (heptanal/heptan-2-one), presented a reversed trend. 

### 2.3. Odor Implications

Some groups of scientists relate the odor contribution of a certain VOC to the human perceivable odor not only to be related to its concentration, but also to its OT [8,9,36,39]. This approach allows some ranking of the VOCs in terms of their relevance to the odor. When the odor activity value (OAV, the average concentration of the volatile compound of the oils divided by its OT) of the volatile compound is greater than one, the odor of this compound is expected to contribute to the odor of the oils according to this theory [53]. In this study, we looked into the odor relevance of compounds using the OTs.

Considering the average concentrations of the identified C1–C4 compounds in OOs and their OTs in Table 1, the odor of those compounds (acetaldehyde, OAV = 185; 2-propenal, OAV = 19; propanal, OAV = 24; acetic acid, OAV = 43; dimethyl sulfide, OAV = 16; propanoic acid, OAV = 12; dimethyl sulfoxide, OAV = 68; butanoic acid, OAV = 143. OAV is calculated based on the average concentration for the OO grade with lowest intensity and its OT in Table 1) are considered to contribute strongly to the odor of the oils due to their high OAVs. Surprisingly, most of those compounds are associated with odor defects.

Regarding the identified C5 compounds, trans-2-pentenal (*m/z* 85.064), associated with green-fruity odor note [1,9], was present with an OAV of 16 for EVOO. Regarding the identified C6 compounds, trans-2-hexenal (*m/z* 99.081), associated with a green-fruity odor note [1,9], was present with an OAV of 13000 for EVOO. Hexanal (*m/z* 101.095), associated with a green-sweet odor note [9], was present with an OAV of 570 for EVOO. Butyl acetate, ethyl butyrate, and ethyl isobutyrate (*m/z* 117.091), associated with a green-sweet-fruity note [1,9], were present with OAVs of 23, 82, and 131 for EVOO, respectively. Therefore, trans-2-pentenal, trans-2-hexenal, hexanal, butyl acetate, ethyl butyrate, and ethyl isobutyrate might be the relevant contributors to the green odor notes of EVOO. This is in agreement with previous studies [9,10] reported that the C5-C6 compounds were described as the most important VOCs in terms of the contribution to the green odor notes for EVOO. Moreover, trans-2-hexenal and hexanal are most likely the most important contributors to the green odor notes of the EVOO due to the highest OAV (13000 and 570, respectively) compared to the other compounds. These results agree with those in a previous study, which reported that the identified C6 aldehydes (especially trans-2-hexenal and hexanal) contribute to the green odor notes in European EVOO [37]. In addition, the OAVs of those compounds mentioned above for EVOO were higher than its lower grade counterparts. Furthermore, it is reported that a great amount of the VOCs associated with the green odor notes have been found in high-quality/grade OO (EVOO) [3,54]. Therefore, the odor of those compounds most likely contributes to the differences in perception of the green odor notes between the premium grade EVOO and the lower grades of OOs.

Although the identified C7–C15 compounds have relatively low concentrations in OOs compared to the identified C1–C6 compounds, they were also components of the volatile odor fraction in OOs, especially the identified C7–C10 compounds [10]. The OAVs of some of these compounds were over one in OOs (2,4 heptadienal, OAV = 3; trans-2-heptenal, OAV = 1.37; heptanal, OAV = 268; heptan-2-one, OAV = 51; trans-2-octenal, OAV = 17; 3-octen-2-one, OAV = 17; octanal, OAV = 338; 1-octen-3-ol, OAV = 27; octan-2-one, OAV = 1.00; trans,trans-2,4-nonadienal, OAV = 75; trans-2-nonenal, OAV = 150; nonanal, OAV = 31; nonan-2-one, OAV = 3; trans,trans-2,4-decadienal, OAV = 8; trans-2-decenal, OAV = 5; decanal, OAV = 7). However, most of those compounds are associated with odor defects. Hexyl acetate (*m/z* 145.122), associated with a green-fruity note, was present with an OAV less than one in OOs, which support previous research [55]. This indicates that this compound might not be a relevant contributor to the green odor notes of OOs. Thus, those minor compounds are more likely related to odor defects of OOs due to their high OAV value and related odor notes.

Summarizing, the identified C5–C6 compounds mainly possess the green odor notes, while the identified C1–C4 and C7–C15 compounds are mainly associated with odor defects. EVOO has 31 volatile compounds exceeding an OAV of one, which is more than ROO (30 volatile compounds) and POO (26 volatile compounds). EVOO is also present with higher OAV values for 29 out of these 31 compounds compared to ROO and POO. Thus, most likely, these VOCs contribute to the richer and more complex odor of EVOO compared to ROO and POO. This is similar to the result in Section 2.2 that EVOO were present with significantly higher headspace concentrations of the VOCs in comparison to ROO and POO.

**Figure 2:**
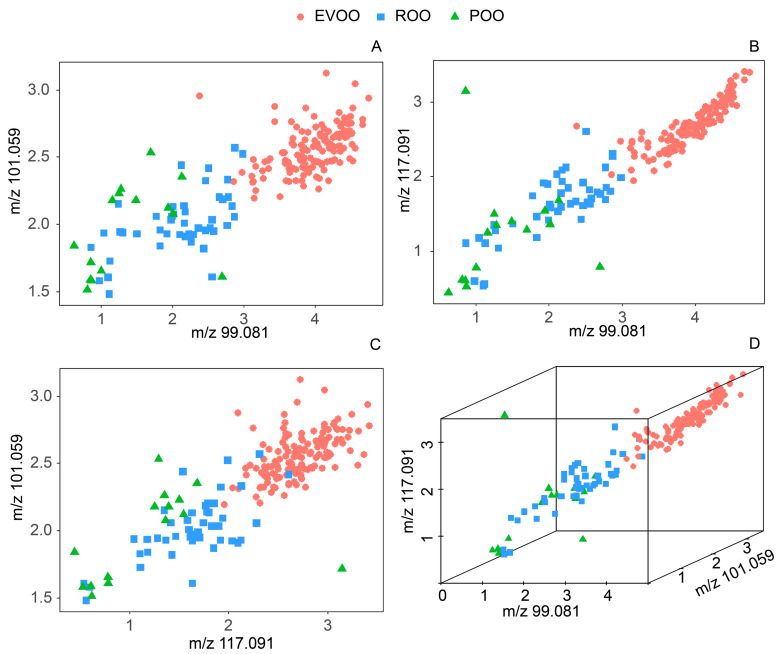
Scatter plots of the ^10^log transformed concentrations (ppbv) of (**A**) *m/z* 99.081 versus *m/z* 101.095; (**B**) *m/z* 99.081 versus *m/z* 117.091; (**C**) *m/z* 101.095 versus *m/z* 117.091; (**D**) *m/z* 99.081, *m/z* 101.095 and *m/z* 143.107 of three olive oil grades, including extra virgin olive oil (EVOO, *n* = 140), refined olive oil (ROO, *n* = 45), and pomace olive oil (POO, *n* = 15).

Consumers’ preference in OOs is mainly related to the odor descriptors qualified with the ‘green’ note [56]. Therefore, the green notes are fairly important sensory traits. In Table 1, trans-2-hexenal (*m/z* 99.081), hexanal (*m/z* 101.095), butyl acetate, ethyl butyrate, and ethyl isobutyrate (*m/z* 117.091) are expected to contribute to the green odor notes of OOs, since their OAVs are greater than one. In order to compare the full sets of samples, the scatter plots of the ^10^log transformed concentrations of *m/z* 99.081, *m/z* 101.095, and *m/z* 117.091 are presented for all samples in Figure 2. The plots show distinct clustering of the three grades of OOs. EVOO (located in the upper right corner in Figure 2) grouped separately from the lower grades of OOs (widely spread in the lower left corner). This indicates that EVOO was present with consistently higher concentrations of these compounds with green notes, and with OAV values >1, in the headspace of EVOO, which is in agreement with previous studies [3,54].

### 2.4. Relative Concentration Differences of the VOCs

To explore the VOCs proportions of OO grades, the relative average concentrations and standard deviation of 54 tentatively identified VOCs in the headspace of each grade (EVOO, ROO, and POO) are shown in Table 2.

Significant differences in the relative concentrations (proportions) in OOs headspace were observed in 50 out of 54 tentatively identified VOCs in Table 2. The major constituents of the VOCs obtained from the headspace of EVOO were identified as methanol (*m/z* 33.033), acetaldehyde (*m/z* 45.034), 2-propenal (*m/z* 57.033), esters (*m/z* 43.018), and ethanol (*m/z* 47.049) in descending order. The VOCs proportions were different in the headspace of the lower grades of OOs. The proportions of the top five major compounds in the ROO headspace were the same as those in the EVOO headspace, except that ethanol changes to acetic acid. However, the top five major compounds in the POO headspace in descending order are acetaldehyde (*m/z* 45.034), formic acid (*m/z* 47.012), methanol (*m/z* 33.033), propanal/acetone (*m/z* 59.049), and acetic acid (*m/z* 61.028).

The relative concentrations of five identified C1–C4 compounds (esters, acetaldehyde, ethanol, acetone, and butan-2-one) in the headspace of ROO/POO were significantly higher than for EVOO (Table 2). Whereas the relative concentrations of the other compounds (2-propenal, acetic acid, dimethyl sulfide, propanoic acid, dimethyl sulfoxide, ethyl acetate, and butanoic acid) in the headspace of ROO/POO were lower than for EVOO (Table 2). Furthermore, 9 out of 11 identified C5–C6 compounds were present in significantly higher relative concentrations for EVOO than for ROO/POO. However, significantly lower proportions were presented in two mass peaks *m/z* 101.095 and *m/z* 103.075 (non-identified) for POO compared to EVOO and ROO. Regarding the identified C7–C15 compounds, 29 out of 30 compounds were present in significant differences in OOs in Table 2. Only 11 out of 29 compounds were present in significantly higher proportions in the EVOO headspace than for the ROO/POO headspace. The relative concentrations of five mass peaks *m/z* 109.101 (methyl norbornene), *m/z* 115.111 (heptanal/heptan-2-one), *m/z* 127.111 (trans-2-octenal/6-methyl-5-hepten-2-one/1-octen-3-one/3-octen-2-one), *m/z* 129.127 (octanal/6-methyl-5-hepten-3-ol/1-octen-3-ol/octan-2-one), and *m/z* 183.082 (benzophenone) in the headspace of ROO and POO were significantly higher than for EVOO. This is different from the concentration results observed in Section 2.2.2 that the other four mass peaks (*m/z* 47.012, *m/z* 49.012, *m/z* 49.027 and *m/z* 115.111) were present in significantly higher concentrations in the headspace of ROO/POO compared to EVOO.

In total, the VOCs proportions were different in the headspace of different OO grades, which is most likely due to the different processing methods. It is known from the literature that absolute concentrations of the VOCs are important for the odor traits of products, but the balance of the VOCs is just as important [57].

## 3. Materials and Methods 

### 3.1. Samples Preparation

For this study, 240 OOs were gathered from producers, traders, and retailers across Europe in 2016 and 2017. The authenticity of the 180 EVOO samples was verified by fatty acid fingerprints combined with chemometrics [58], ultraviolet-visible spectra analysis [59], and evaluated by 2/3-monochloropropane-1,2-diol and glycidyl esters analysis [60]. A total of 40 EVOO samples did not meet the requirements for one or more of these methods and were therefore removed from the sample set. In total, 200 OOs were used in this study, which consisted of 140 EVOO, 45 ROO, and 15 POO. Prior to analysis, all the oils were sealed and stored in the dry and dark environment at 18 ± 1 °C. Sampling was completed within 6 months. To avoid long-term storage, the analyses were carried out within two weeks after sampling of each sample.

The sample preparation method was similar to our previous study [25] with minor modifications. Firstly, 5 mL of oil was transferred into a 250 mL flask. Then, the closed flask was kept in a water bath at 30 °C for 30 min to equilibrate the headspace before instrumental analysis. 

### 3.2. PTR-QiToF-MS Analysis

PTR-QiToF-MS (Ionicon Analytik GmbH, Innsbruck, Austria) was operated with a drift voltage of 999 V, a drift temperature of 61 ± 1 °C, a drift pressure of 3.803 mbar, and an E/N value of 134 ± 1 Townsend. The laboratory air was measured for the first 10 s as a blank before each sample. Then, the VOCs in the headspace of the flask were transported to the PTR-QiToF-MS through a peek inlet tube with a temperature of 60 ± 0.5 °C. The flow rate of the air in the tube was 61 ± 2 mL min^−1^. The measurement time was 30 s. The acquisition rate was 1 spectrum per second. On each of two different days, one independent replicates per sample was measured. Samples were analyzed in a random sequence to avoid any order bias. Results were stored in the system automatically.

### 3.3. VOCs Data Pre-Processing

All the raw data obtained from the PTR-QiToF-MS machine were integrated by PTRwid software (Utrecht University, Utrecht, the Netherlands; http://www.staff.science.uu.nl/~holzi101/ptrwid/) [61]. The unified mass list with the ion count per second (cps) of each sample were provided after the autonomous mass scale calibration, as described by Holzinger [61]. The average of the 30 sample scans and the average of the 10 blank scans were calculated separately. The VOC concentrations (molecules per cm^3^) were calculated from cps according to Equation (1) [62]:

(1) $$\left[VOC\right]=\frac{1}{kt}\times \frac{{\left[VOC\cdot H^{+}\right]}_{measured}}{{\left[H_{3}O^{+}\right]}_{measured}\times 487}\times \frac{\sqrt{{\left(m/z\right)}_{H_{3}O^{+}}}}{\sqrt{{\left(m/z\right)}_{VOC\cdot H^{+}}}}$$

where t is the residence time of the primary ions in the drift tube, k is the coefficient of the reaction rate with a value of 2 × 10^−9^ cm^3^/s, [VOC·H^+^]_measured_ is the ion count rate of the protonated VOC, [H_3_O^+^]_measured_ is the ion count rate of the protonated water at *m/z* 21.022, 487 is the intensity ratio of the protonated water at *m/z* 19.018 (100%) to the protonated water at *m/z* 21.022 (0.2055%) [63], (*m/z*)_H3O+_ and (*m/z*)_VOC·H+_ are the molecular weight of protonated water and protonated VOC.

Subsequently, the unit of molecules per cm^3^ was converted to ppbv, on the basis of ideal gas using Equation (2) [64]:

(2) PV=nRT

where P (Pa) is the pressure, V (cm^3^) is the volume, n (mol) is the number of moles, R (J K^−1^/mol) is the gas constant, and T (K) is the temperature.

After unit conversion, the average of each sample’s 10 blank cycles were subtracted from the sample’s averaged scan. The replicates of each sample were checked using autocorrelation [65], and the sample will be removed when the correlation value was below 0.9. In this study, the correlation values of all the samples were over 0.9. Finally, sample averages were calculated from the data of two replicate measurements.

Sample independent ions, such as N_2_^+^, NO^+^, O_2_^+^, H_2_O^+^, H_3_^18^O^+^, (H_2_O)_2_∙H^+^, H_2_O∙H_2_^18^O∙H^+^ and (H_2_O)_3_∙H^+^] signals at mass peaks *m/z* 28.005, *m/z* 29.997, *m/z* 31.989, *m/z* 18.010, *m/z* 21.022, *m/z* 37.028, *m/z* 39.032, and *m/z* 55.039, respectively, were removed. After the data pre-processing, 295 mass peaks in the range of *m/z* 18.033 to 207.204 remained.

### 3.4. Relative Concentration

The relative concentration (C, %) of each mass of each sample was calculated by the intensity of the single mass (I_s_, ppbv) per sample and the total mass intensity (I_t_, ppbv) per sample using Equation (3):

(3) $$C=\frac{I_{s}}{I_{t}}\times 100\%.$$

### 3.5. Data Analysis

Significant differences of the (relative) concentrations of tentatively identified VOCs for different grades of OOs were assessed using non-parametric Kruskal–Wallis tests (*p* < 0.05). Mann–Whitney U-tests were used to perform pairwise comparisons between OO grades. These analyses methods were performed using SPSS (version 23, IBM, Chicago, IL, USA).

### 3.6. Odor Threshold in Air

The OTs of the identified compounds were collected from several publications [29,39,66,67,68,69]. Firstly, the OT in oil were converted into the OT in air using Equation (4):

(4) $$C_{a1}=KC_{o}\rho$$

where C_a1_ (µg/L) is the OT in air, C_o_ (µg/kg) is the OT in oil, K is the air/liquid partition coefficient of compound [67], and ρ is the density of olive oil (0.916 kg/L).

Then, the unit of the OT in air was converted using Equation (5) [64]:

(5) $$C_{a2}=V_{m}\frac{C_{a1}}{M}\times 1000$$

where C_a2_ (ppbv) is the OT in air, V_m_ is the standard molar volume of ideal gas at 1 bar and 298 K with a value of 24.77 L/mol [70], and M (g/mol) is the molecular weight of the compound.

Subsequently, the lowest OT of each compound was used in this study. The calculated OTs in air from various literature sources are provided in the Supplementary Material (Table S1). The OAV was calculated using the average concentration of the volatile compound for each of the type of OO divided by the corresponding OT.

## 4. Conclusions

Significant differences in VOC headspace concentrations were determined for the different grades of OOs. Most of the VOCs were present in significantly higher concentrations for EVOO than for ROO/POO. However, significantly higher concentrations of mass peaks *m/z* 47.012 (formic acid), *m/z* 49.016, *m/z* 49.027, and *m/z* 115.111 (heptanal/heptanone) were found for the lower grades of OOs (ROO/POO) compared to EVOO. Furthermore, significant differences of the VOCs proportions were observed indicating a distinct change in the balance of the VOCs across OO grades. Thus, EVOO and ROO/POO not only differ quantitatively (concentrations of compounds) but also qualitatively (proportions of compounds). Comparison with OAVs of the compounds revealed the expected change in contribution to the odor of the OOs. Our results underpin the well-known richer and more complex odor of EVOO by the elevated contribution of the VOCs with green notes exceeding the minimal OAV. Furthermore, the consistent differences in VOCs concentrations between EVOO and other grades of OO may provide potential for verification of the identity of OOs.

## Figures and Tables

**Table 1:** Tentatively identified volatile organic compounds of extra virgin olive oil (EVOO, n = 140), refined olive oil (ROO, n = 45), and pomace olive oil (POO, n = 15), their odor notes, odor thresholds (OT, ppbv) in air, average headspace concentrations (ppbv), standard deviation (SD), and statistical comparisons (Kruskal–Wallis tests and Mann–Whitney U-tests, *p* < 0.05). ppbv: parts per billion by volume.

Measured Protonated Mass *m/z*	Protonated Chemical Formula	Tentative Identification	Type	Reference	Odor Notes	Reference	OT	Average ± SD (ppbv)		
								EVOO	ROO	POO
**C1–C4 ^A^**										
33.033	CH_5_O^+^	Methanol	Alcohols	[27,28]	Sweet	[29]	100 × 10^3^	(348 ± 98) × 10^3 a^	(106 ± 93) × 10^3 b^	(18 ± 37) × 10^3 b^
43.018	C_2_H_3_O^+^	Esters	Esters	[30]	Ester, green, pungent, sweet, fruity	[30]		(44 ± 40) × 10^3 a^	(16 ± 18) × 10^3 b^	(6 ± 4) × 10^3 b^
45.034	C_2_H_5_O^+^	Acetaldehyde	Aldehydes	[9]	Pungent, sweet	[9]	210	(64 ± 39) × 10^3 a^	(41 ± 28) × 10^3 b^	(39 ± 34) × 10^3 ab^
47.012	CH_3_O_2_^+^	Formic acid	Carboxylic acids	[31,32]	Pungent, penetrating	[33]	28 × 10^3^	(3 ± 3) × 10^3 b^	(12 ± 11) × 10^3 a^	(17 ± 16) × 10^3 a^
47.049	C_2_H_7_O^+^	Ethanol	Alcohols	[1,9]	Apple, sweet, alcohol	[1,9]	10 × 10^3^	(45 ± 55) × 10^3 a^	(18 ± 25) × 10^3 b^	(4 ± 8) × 10^3 b^
57.033	C_3_H_5_O^+^	2-Propenal	Aldehydes	[32]	Unpleasant odor, irritating	[34,35]	210	(39 ± 33) × 10^3 a^	(11 ± 12) × 10^3 b^	(4 ± 5) × 10^3 b^
59.049	C_3_H_7_O^+^	Propanal	Aldehydes	[28,36]	Pungent, sweet	[36]	419	(16 ± 12) × 10^3 a^	(12 ± 9) × 10^3 b^	(10 ± 7) × 10^3 b^
		Acetone	Ketones	[28]	Sweet, pungent	[29]	100 × 10^3^			
61.028	C_2_H_5_O_2_^+^	Acetic acid	Carboxylic acids	[9,37]	Sour, vinegary	[9]	162	(33 ± 33) × 10^3 a^	(20 ± 24) × 10^3 ab^	(7 ± 5) × 10^3 b^
63.026	C_2_H_7_S^+^	Dimethyl sulfide	Others	[30,38]	Wet earth, organic, beetroot, sulfury	[30,38]	1	(1 ± 2) × 10^3 a^	45 ± 54 ^b^	16 ± 11 ^b^
73.064	C_4_H_9_O^+^	Butan-2-one	Ketones	[9]	Ethereal, fruity	[9]	10 × 10^3^	(4 ± 3) × 10^3 a^	(3 ± 2) × 10^3 a^	(3 ± 2) × 10^3 b^
75.044	C_3_H_7_O_2_^+^	Propanoic acid	Carboxylic acids	[9,32,39]	Pungent, sour, mold	[9,39]	33	(22 ± 21) × 10^3 a^	(3 ± 3) × 10^3 b^	391 ± 471 ^b^
		Methyl acetate	Esters	[1]	Ethereal, sweet	[1]	561 × 10^3^			
79.021	C_2_H_7_OS^+^	Dimethyl sulfoxide	Others	[37,40]	Unpleasant	[37]	1	711 ± 810 ^a^	197 ± 183 ^b^	68 ± 51 ^b^
89.059	C_4_H_9_O_2_^+^	Ethyl acetate	Esters	[1,9]	Sticky, sweet, ethereal	[1,9]	1 × 10^3^	(4 ± 4) × 10^3 a^	(1 ± 1) × 10^3 b^	143 ± 236 ^b^
		Butanoic acid	Carboxylic acids	[9,39]	Rancid, cheese	[9,39]	1			
**C5**										
85.064	C_5_H_9_O^+^	trans-2-Pentenal; trans-2-methyl-2-butenal	Aldehydes	[1,9]	Green, apple, bitter almond; green fruity, aromatic	[1,9]	437 ^B^	(7 ± 6) × 10^3 a^	284 ± 204 ^b^	105 ± 109 ^b^
		1-Penten-3-one	Ketones	[1,9,37]	Green, pungent, mustard	[1,9]				
87.081	C_5_H_11_O^+^	3-Penten-2-ol; 2-methyl-3-buten-2-ol	Alcohols	[1,9]	Perfumery, woody; grassy, earth, oily	[1,9]		(13 ± 8) × 10^3 a^	(1 ± 1) × 10^3 b^	319 ± 208 ^b^
		2-Methylbutanal; 3-methylbutanal; pentanal	Aldehydes	[9,37]	Malty; malty; woody, bitter, oily	[9]	11 ^C^			
		3-Pentanone	Ketones	[30,40]	Sweet, green	[30]				
103.075	C_5_H_11_O_2_^+^	Ethyl propionate	Esters	[1,37]	Strawberry, apple, fruity, sweet	[1]	2 × 10^3^	208 ± 98 ^a^	69 ± 77 ^b^	91 ± 252 ^b^
		3-Methylbutanoic acid; pentanoic acid	Carboxylic acids	[9,32,37,39]	Sweaty; unpleasant, pungent	[9,39]	19; 9			
**C6**										
81.070	C_6_H_9_^+^	Terpene fragment or fragments from cis-/trans-hexenal	Others	[41,42]	-	-		(12 ± 12) × 10^3 a^	337 ± 280 ^b^	74 ± 68 ^b^
83.085	C_6_H_11_^+^	Terpene fragment	Others	[41]	-	-		(8 ± 3) × 10^3 a^	(1 ± 1) × 10^3 b^	(1 ± 1) × 10^3 b^
85.101	C_6_H_13_^+^	Cyclohexane	Others	[43]	Sweet, aromatic	[33]	728 × 10^3^	(3 ± 2) × 10^3 a^	259 ± 220 ^b^	110 ± 89 ^b^
97.064	C_6_H_9_O^+^	2,4-Hexadienal	Aldehydes	[1]	Fresh, green, floral, citric	[1]		277 ± 159 ^a^	25 ± 14 ^b^	20 ± 17 ^b^
		Ethyl furan	Others	[1,43]	Sweet, ethereal	[1,43]				
99.081	C_6_H_11_O^+^	trans-2-Hexenal; cis-3-hexenal	Aldehydes	[1,9,37]	Green, apple, bitter almonds, astringent; green, leaf-like	[1,9]	1 ^D^	(13 ± 11) × 10^3 a^	243 ± 240 ^b^	66 ± 125 ^b^
101.095	C_6_H_13_O^+^	cis-3-Hexen-1-ol; trans-3-hexen-1-ol; cis-2-hexen-1-ol; trans-2-hexen-1-ol	Alcohols	[1,9,10,37]	Green; green grassy, sweet; almond, grassy, astringent; green grassy, leaves, fruity, astringent, bitter	[1,9]		154 ± 78 ^a^	57 ± 35 ^b^	35 ± 31 ^b^
		Hexanal; 3-methyl pentanal	Aldehydes	[1,9,37]	Green-sweet, green-apple, grassy;	[9]	0.27 ^E^			
		4-Methylpentan-2-one	Ketones	[1,43]	Strawberry, fruity, sweet, ethereal	[1,43]	470			
113.059	C_6_H_9_O_2_^+^	Sorbic acid	Carboxylic acids	[18]	-	-		171 ± 308 ^a^	10 ± 7 ^b^	5 ± 4 ^b^
		5-Ethyl-2-(5H)-furanone	Ketones	[40]	-	-				
117.091	C_6_H_13_O_2_^+^	Butyl acetate; ethyl butyrate; ethyl isobutyrate	Esters	[1,9,37]	Green, fruity, pungent; sweet, fruity, cheesy; fruity	[1,9]	29; 8; 5	657 ± 488 ^a^	66 ± 69 ^b^	110 ± 358 ^b^
		Hexanoic acid	Carboxylic acids	[9,39]	Pungent, rancid, sour, sharp	[9,39]	127			
**C7–C15**										
93.070	C_7_H_9_^+^	Toluene	Others	[30,37,40]	Gasoline vapors	[30,37]	2 × 10^3^	254 ± 223 ^a^	33 ± 51 ^b^	7 ± 12 ^b^
105.090	C_8_H_9_^+^	Ethenyl benzene	Others	[1,30]	Gasoline vapors	[30]	47	324 ± 247 ^a^	48 ± 49 ^b^	18 ± 13 ^b^
107.086	C_8_H_11_^+^	Ethyl benzene	Others	[1]	Strong	[1]	39 × 10^3^	338 ± 275 ^a^	75 ± 131 ^b^	17 ± 27 ^b^
109.101	C_8_H_13_^+^	Methyl norbornene	Others	[18]	-	-		50 ± 24 ^a^	29 ± 24 ^b^	33 ± 20 ^ab^
111.080	C_7_H_11_O^+^	2,4-Heptadienal	Aldehydes	[9,39]	Fatty, rancid, nutty	[9,39]	8	86 ± 62 ^a^	38 ± 32 ^b^	27 ± 34 ^b^
113.096	C_7_H_13_O^+^	trans-2-Heptenal	Aldehydes	[9]	Oxidized, tallowy, pungent	[9]	19	131 ± 125 ^a^	30 ± 22 ^b^	26 ± 30 ^b^
115.111	C_7_H_15_O^+^	Heptanal	Aldehydes	[9,10,39]	Oily, fatty, woody, rancid	[9,39]	0.19	51 ± 31 ^b^	81 ± 65 ^a^	77 ± 63 ^ab^
		Heptan-2-one	Ketones	[1,9]	Sweet, fruity, cinnamon	[1,9]	1			
121.099	C_9_H_13_^+^	1,2,4-Trimethylbenzene	Others	[1,10]	-	-	25 × 10^3^	78 ± 59 ^a^	7 ± 13 ^b^	2 ± 5 ^b^
	C_8_H_9_O^+^	Acetophenone	Ketones	[40]	-	-				
123.080	C_8_H_11_O^+^	2-Phenylethanol	Alcohols	[10,40]	-	-		4 ± 2 ^a^	1 ± 2 ^b^	1 ± 1 ^b^
125.096	C_8_H_13_O^+^	cis-1,5-Octadien-3-one; octan-2-one	Ketones	[1,9,37]	Geranium-like; mold, overripe	[1,9]		34 ± 26 ^a^	13 ± 11 ^b^	5 ± 5 ^b^
		trans,trans-2,4-Octadienal	Aldehydes	[10]	-	-				
127.111	C_8_H_15_O^+^	trans-2-Octenal	Aldehydes	[1,9]	Herbaceous, spicy	[1,9]	1	37 ± 17 ^a^	17 ± 12 ^b^	19 ± 13 ^b^
		6-Methyl-5-hepten-2-one; 1-octen-3-one; 3-octen-2-one	Ketones	[1,9,10,37]	Pungent, green fruity, grassy; mushroom, mold, pungent; rose	[1,9,44]	1 ^F^			
129.091	C_7_H_13_O_2_^+^	3-Methyl-2-butenyl acetate	Esters	[1]	Pungent	[1]		16 ± 8 ^a^	5 ± 4 ^b^	5 ± 5 ^b^
129.127	C_8_H_17_O^+^	Octanal	Aldehydes	[9,37,39]	Fatty, sharp, citrus-like, rancid	[9,39]	0.08	27 ± 15	41 ± 36	59 ± 81
		6-Methyl-5-hepten-3-ol; 1-octen-3-ol	Alcohols	[9,39]	Perfumery, nutty, perfumery, nutty; mold, earthy	[9,39]	1 ^G^			
		Octan-2-one	Ketones	[9]	Mold, green	[9]	27			
131.106	C_7_H_15_O_2_^+^	Propyl butyrate; ethyl 2-methylbutyrate; ethyl 3-methylbutyrate	Esters	[1,9,37]	Pineapple, sharp; fruity; fruity, green, banana	[1,9]	2 × 10^3 H^	72 ± 52 ^a^	21 ± 28 ^b^	6 ± 6 ^b^
		Heptanoic acid	Carboxylic acids	[9,39]	Rancid, fatty	[9,39]				
137.132	C_10_H_17_^+^	Terpene fragments (α-pinene; β-pinene; limonene; tricyclene; camphene; sabinene; myrcene; β-ocimene)	Others	[10,32]	-	-	3 × 10^3^; 6 × 10^3^; 130 ^I^	89 ± 68 ^a^	37 ± 70 ^b^	12 ± 18 ^b^
139.112	C_9_H_15_O^+^	trans,trans-2,4-Nonadienal	Aldehydes	[9,37]	Soapy, penetrating, deep-fried	[9]	0.04	19 ± 12 ^a^	3 ± 1 ^b^	5 ± 3 ^b^
139.147	C_10_H_19_^+^	3-Ethyl-1,5-octadiene	Others	[40]	-	-		82 ± 50 ^a^	6 ± 5 ^b^	4 ± 3 ^b^
141.127	C_9_H_17_O^+^	tran-2-Nonenal; cis-2-nonenal	Aldehydes	[9,36,37]	Paper-like, fatty; green, fatty	[9,36]	0.02 ^J^	8 ± 8 ^a^	3 ± 3 ^b^	5 ± 4 ^a^
143.107	C_8_H_15_O_2_^+^	cis-3-Hexenyl acetate; trans-3-hexenyl acetate; 3-methyl-4-penten-1-ol-acetate	Esters	[1,9,10,37]	Green, banana-like; green, banana, green leaves, fruity	[1,9]		49 ± 25 ^a^	8 ± 7 ^b^	3 ± 3 ^b^
143.142	C_9_H_19_O^+^	Nonanal	Aldehydes	[9,10,37]	Fatty, waxy, pungent	[9]	0.45	44 ± 23 ^a^	17 ± 11 ^b^	14 ± 12 ^b^
		Nonan-2-one	Ketones	[1]	Fruity, floral	[1]	5			
145.122	C_8_H_17_O_2_^+^	Hexyl acetate; 2-methylpropyl butanoate; ethyl hexanoate	Esters	[1,9,10]	Green, fruity, sweet, apple; unpleasant, winey, fusty	[1,9]	307 ^K^	58 ± 36 ^a^	8 ± 9 ^b^	4 ± 3 ^b^
		Octanoic acid	Carboxylic acids	[9,39]	Oily, fatty	[9,39]				
153.125	C_10_H_17_O^+^	2,4-Decadienal; trans, trans-2,4-decadienal; trans, cis-2,4-decadienal	Aldehydes	[9,37]	Strong, fatty; deep-fried; deep-fried	[9]	0.37 ^L^	8 ± 3 ^a^	3 ± 3 ^b^	3 ± 3 ^b^
		2,3-Dehydro-1,8-cineole	Others	[10]	-	-				
155.141	C_10_H_19_O^+^	trans-2-Decenal	Aldehydes	[9]	Painty, fishy, fatty	[9]	0.43	6 ± 3 ^a^	3 ± 3 ^b^	2 ± 4 ^b^
		3,7-Dimethylocta-1,6-dien-3-ol; cis-p-menth-2-en-1-ol	Alcohols	[10]	-	-				
157.124	C_9_H_17_O_2_^+^	Ethyl cyclohexanecarboxylate	Esters	[9,37]	Aromatic, fruity	[9]		11 ± 6 ^a^	1 ± 1 ^b^	1 ± 1 ^b^
157.158	C_10_H_21_O^+^	Decanal	Aldehydes	[9,10,43]	Penetrating, sweet, waxy, painty	[9,43]	0.41	5 ± 3 ^a^	3 ± 3 ^b^	4 ± 6 ^ab^
169.123	C_10_H_17_O_2_^+^	trans-4,5-Epoxy-trans-2-decenal	Aldehydes	[9,10,37]	Metallic	[9]		2 ± 2 ^a^	0.5 ± 0.4 ^b^	0.3 ± 0.3 ^b^
171.174	C_11_H_23_O^+^	Undecanal	Aldehydes	[43]	Fatty, tallowy	[43]		3 ± 1 ^a^	0.4 ± 0.4 ^b^	0.3 ± 0.4 ^b^
173.154	C_10_H_21_O_2_^+^	Ethyl octanoate; methyl nonanoate	Esters	[32,36,37]	Green, fruity; -	[36]		11 ± 24 ^a^	1 ± 1 ^b^	0.8 ± 0.9 ^b^
183.082	C_13_H_11_O^+^	Benzophenone	Others	[40]	-	-		2 ± 2 ^a^	0.8 ± 0.4 ^b^	0.6 ± 0.3 ^b^
205.194	C_15_H_25_^+^	β-Caryophyllene; copaene; β-selinene; α-farnesene; eremophilene	Others	[10,32,40]	-	-		23 ± 22 ^a^	4 ± 7 ^b^	0.4 ± 0.7 ^b^

Superscript letters a and b in a row indicate significant differences (*p* < 0.05). ^A^ C1–C7 indicate that the VOCs with 1–7 carbon atoms in the molecule, respectively. ^B^ The OT of trans-2-pentenal. ^C^ The OT of pentanal. ^D^ The OT of trans-2-hexenal. ^E^ The OT of hexanal. ^F^ The OT of 3-octen-2-one. ^G^ The OT of 1-octen-3-ol. ^H^ The OT of propyl butyrate. ^I^ 2981, 5465, 119 refer to the OTs of α-pinene, β-pinene, limonene, respectively. ^J^ The OT of tran-2-nonenal. ^K^ The OT of hexyl acetate. ^L^ 2,4-Decadienal. Green indicates that the odor activity value (OAV, the average concentration of the volatile compound of the oils divided by its OT) is more than 2. Yellow indicates that the OAV is between 1 and 2. Red indicates that the OAV is less than 1. Rows without color indicate that no OT was found for this compound.

**Table 2:** Tentative identification volatile organic compounds (VOCs), average relative concentrations, standard deviation (SD), and statistical comparisons (Kruskal–Wallis tests and Mann–Whitney U-tests, *p* < 0.05) of the VOCs in the headspace of extra virgin olive oil (EVOO), refined olive oil (ROO), and pomace olive oil (POO). The relative concentration of each mass per sample was expressed as the ratio (%, *w*/*w*) of the single mass peak intensity per sample to the total mass intensity per sample.

Measured Protonated Mass *m/z*		Average ± SD (%)		
	Tentative Identification	EVOO (*n* = 140)	ROO (*n* = 45)	POO (*n* = 15)
**C1–C4 ^A^**				
33.033	Methanol	46 ± 9 ^a^	33 ± 14 ^b^	10 ± 10 ^c^
43.018	Esters	5 ± 3 ^b^	6 ± 3 ^a^	5 ± 1 ^ab^
45.034	Acetaldehyde	8 ± 3 ^b^	16 ± 6 ^a^	30 ± 17 ^a^
47.012	Formic acid	(4 ± 5) × 10^−1 b^	5 ± 4 ^a^	14 ± 7 ^a^
47.049	Ethanol	5 ± 5	5 ± 4	3 ± 2
57.033	2-Propenal	6 ± 5	6 ± 8	3 ± 3
59.049	Propanal; acetone	2 ± 2 ^b^	5 ± 3 ^a^	9 ± 3 ^a^
61.028	Acetic acid	4 ± 3 ^b^	7 ± 3 ^a^	6 ± 3 ^a^
63.026	Dimethyl sulfide	(1 ± 2) × 10^−1 a^	(2 ± 2) × 10^−2 b^	(1 ± 0) × 10^−2 b^
73.064	Butan-2-one	1 ± 1 ^b^	2 ± 1 ^a^	3 ± 2 ^a^
75.044	Propanoic acid; methyl acetate	3±2 ^a^	1±0 ^b^	(3 ± 3) × 10^−1 c^
79.021	Dimethyl sulfoxide	(8 ± 7) × 10^−2^	(1 ± 1) × 10^−1^	(6 ± 2) × 10^−2^
89.059	Ethyl acetate; butanoic acid	(4 ± 3) × 10^−1 a^	(2 ± 2) × 10^−1 b^	(2 ± 5) × 10^−1 c^
**C5–C6**				
81.070	Terpene fragment or fragments from cis-/trans-hexenal	2 ± 2 ^a^	(2 ± 1) × 10^−1 b^	(7 ± 5) × 10^−2 b^
83.085	Terpene fragment	1 ± 0 ^a^	1 ± 0 ^b^	1 ± 0 ^b^
85.064	trans-2-Pentenal; trans-2-methyl-2-butenal; 1-penten-3-one	1 ± 1 ^a^	(1 ± 1) × 10^−1 b^	(1 ± 0) × 10^−1 b^
85.099	Cyclohexane	(4 ± 3) × 10^−1 a^	(9 ± 4) × 10^−2 b^	(9 ± 4) × 10^−2 b^
87.081	3-Penten-2-ol; 2-methyl-3-buten-2-ol; 2-methylbutanal; 3-methylbutanal; pentanal; 3-pentanone	2 ± 1 ^a^	(4 ± 2) × 10^−1 b^	(3 ± 1) × 10^−1 b^
97.064	2,4-Hexadienal; ethyl furan	(4 ± 2) × 10^−2 a^	(1 ± 1) × 10^−2 b^	(2 ± 1) × 10^−2 b^
99.081	trans-2-Hexenal; cis-3-hexenal	2 ± 2 ^a^	(1 ± 1) × 10^−1 b^	(1 ± 2) × 10^−1 b^
101.095	cis-3-Hexen-1-ol; trans-3-hexen-1-ol; cis-2-hexen-1-ol; trans-2-hexen-1-ol; hexanal; 3-methyl pentanal; 4-methylpentan-2-one	(5 ± 2) × 10^−2 b^	(6 ± 7) × 10^−2 b^	(1 ± 1) × 10^−1 a^
103.075	Ethyl propionate; 3-methylbutanoic acid; pentanoic acid	(3 ± 1) × 10^−2 b^	(2 ± 2) × 10^−2 b^	(2 ± 6) × 10^−1 a^
113.059	Sorbic acid; 5-ethyl-2-(5H)-furanone	(2 ± 4) × 10^−2 a^	(4 ± 3) × 10^−3 b^	(4 ± 5) × 10^−3 b^
117.091	Butyl acetate; ethyl butyrate; ethyl isobutyrate; hexanoic acid	(9 ± 7) × 10^−2 a^	(2 ± 1) × 10^−2 b^	(2 ± 9) × 10^−1 b^
**C7–C15**				
93.070	Toluene	(3 ± 4) × 10^−2 a^	(1 ± 1) × 10^−2 b^	(1 ± 1) × 10^−2 b^
105.090	Ethenyl benzene	(4 ± 2) × 10^−2 a^	(2 ± 2) × 10^−2 b^	(2 ± 1) × 10^−2 b^
107.086	Ethyl benzene	(4 ± 3) × 10^−2 a^	(2 ± 2) × 10^−2 b^	(2 ± 5) × 10^−2 b^
109.101	Methyl norbornene	(1 ± 0) × 10^−2 c^	(1 ± 1) × 10^−2 b^	(3 ± 2) × 10^−2 a^
111.080	2,4-Heptadienal	(1 ± 1) × 10^−2 b^	(2 ± 1) × 10^−2 a^	(2 ± 1) × 10^−2 ab^
113.096	trans-2-Heptenal	(2 ± 1) × 10^−2 ab^	(1 ± 1) × 10^−2 b^	(2 ± 1) × 10^−2 a^
115.111	Heptanal; heptan-2-one	(1 ± 0) × 10^−2 b^	(6 ± 1) × 10^−2 a^	(1 ± 1) × 10^−1 a^
121.099	1,2,4-Trimethylbenzene; acetophenone	(9 ± 6) × 10^−3 a^	(2 ± 3) × 10^−3 b^	(3 ± 11) × 10^−3 b^
123.080	2-Phenylethanol	(6 ± 2) × 10^−4 a^	(5 ± 6) × 10^−4 b^	(9 ± 8) × 10^−4 a^
125.096	cis-1,5-Octadien-3-one; octan-2-one; trans, trans-2,4-octadienal	(5 ± 3) × 10^−3^	(6 ± 5) × 10^−3^	(4 ± 2) × 10^−3^
127.111	trans-2-Octenal; 6-methyl-5-hepten-2-one; 1-octen-3-one; 3-octen-2-one	(5 ± 3) × 10^−3 c^	(9 ± 7) × 10^−3 b^	(2 ± 1) × 10^−2 a^
129.091	3-Methyl-2-butenyl acetate	(2 ± 1) × 10^−3 ab^	(3 ± 5) × 10^−3 b^	(6 ± 9) × 10^−3 a^
129.127	Octanal; 6-methyl-5-hepten-3-ol; 1-octen-3-ol; octan-2-one	(4 ± 2) × 10^−3 b^	(3 ± 6) × 10^−2 a^	(9 ± 15) × 10^−2 a^
131.106	Propyl butyrate; ethyl 2-methylbutyrate; ethyl 3-methylbutyrate; heptanoic acid	(9 ± 5) × 10^−3 a^	(7 ± 4) × 10^−3 b^	(7 ± 12) × 10^−3 b^
137.132	Terpene fragments (α-pinene; β-pinene; limonene; tricyclene; camphene; sabinene; myrcene; β-ocimene)	(1 ± 1) × 10^−2 b^	(2 ± 8) × 10^−2 a^	(1 ± 2) × 10^−2 ab^
139.112	trans,trans-2,4-Nonadienal	(3 ± 2) × 10^−3 b^	(1 ± 2) × 10^−3 c^	(6 ± 4) × 10^−3 a^
139.147	3-Ethyl-1,5-octadiene	(1 ± 1) × 10^−2 a^	(2 ± 1) × 10^−3 b^	(5 ± 3) × 10^−3 b^
141.127	tran-2-Nonenal; cis-2-nonenal; cis-3-nonenal	(1 ± 1) × 10^−3 b^	(1 ± 1) × 10^−3 b^	(5 ± 2) × 10^−3 a^
143.107	cis-3-Hexenyl acetate; trans-3-hexenyl acetate; 3-methyl-4-penten-1-ol-acetate	(7 ± 5) × 10^−3 a^	(3 ± 3) × 10^−3 b^	(2 ± 1) × 10^−3 b^
143.142	Nonanal; nonan-2-one	(6 ± 4) × 10^−3 b^	(1 ± 1) × 10^−2 ab^	(1 ± 1) × 10^−2 a^
145.122	Hexyl acetate; 2-methylpropyl butanoate; ethyl hexanoate; octanoic acid	(8 ± 4) × 10^−3 a^	(3 ± 1) × 10^−3 b^	(4 ± 5) × 10^−3 b^
153.125	2,4-Decadienal; trans, trans-2,4-decadienal; trans, cis-2,4-decadienal; 2,3-dehydro-1,8-cineole	(1 ± 0) × 10^−3 ab^	(1 ± 1) × 10^−3 b^	(2 ± 3) × 10^−3 a^
155.141	trans-2-Decenal; 3,7-dimethylocta-1,6-dien-3-ol; cis-p-menth-2-en-1-ol	(1 ± 0) × 10^−3 ab^	(1 ± 1) × 10^−3 b^	(2 ± 2) × 10^−3 a^
157.124	Ethyl cyclohexanecarboxylate	(2 ± 1) × 10^−3 a^	(7 ± 8) × 10^−4 b^	(1 ± 1) × 10^−3 a^
157.158	Decanal	(7 ± 5) × 10^−4 b^	(2 ± 4) × 10^−3 b^	(6 ± 10) × 10^−3 a^
169.123	trans-4,5-Epoxy-trans-2-decenal	(3 ± 2) × 10^−4 a^	(2 ± 4) × 10^−4 b^	(3 ± 3) × 10^−4 ab^
171.174	Undecanal	(4 ± 3) × 10^−4 a^	(1 ± 2) × 10^−4 b^	(4 ± 6) × 10^−4 b^
173.154	Ethyl octanoate; methyl nonanoate	(1 ± 3) × 10^−3 a^	(4 ± 3) × 10^−4 b^	(6 ± 5) × 10^−4 b^
183.082	Benzophenone	(4 ± 7) × 10^−4 b^	(4 ± 3) × 10^−4 a^	(7 ± 6) × 10^−4 a^
205.194	β-Caryophyllene; copaene; β-selinene; α-farnesene; eremophilene	(3 ± 3) × 10^−3 a^	(1 ± 1) × 10^−3 b^	(3 ± 3) × 10^−4 c^

Superscript letters a, b, and c indicate the significant differences (*p* < 0.05). **^A^** C1–C7 are the VOCs with 1–7 carbon atoms in the molecule.

## Supplementary Materials

The following are available online, Table S1: List of the air/liquid partition coefficients (K) and the calculated odor thresholds (OTs) in air (ppbv) of volatile organic compounds.
