# Supplementary material for: From Extra Virgin Olive Oil to Refined Products: Intensity and Balance Shifts of the Volatile Compounds versus Odor
Source: Molecules. 2020 May 26;25(11):2469. doi: 10.3390/molecules25112469 (PMC7321329; doi:10.3390/molecules25112469)
Supplement: Supplementary file 1 [file molecules-25-02469-s001.pdf]

Supplementary Materials

From Extra Virgin Olive Oil to Refined Products: Intensity and Balance Shifts of the Volatile Compounds versus Odor

Jing Yan [1,2], Martin Alewijn [2], Saskia M. van Ruth [1,2,*]

[1] Food Quality and Design Group, Wageningen University and Research, P.O. Box 17, 6700AA Wageningen, The Netherlands; jing.yan@wur.nl

[2] Wageningen Food Safety Research, Wageningen University and Research, P.O. Box 230, 6700 AE Wageningen, The Netherlands; martin.alewijn@wur.nl

* Correspondence: saskia.vanruth@wur.nl; Tel.: +31-317-480250

Table S1. List of the air/liquid partition coefficients (K) and the calculated odor thresholds (OTs) in air (ppbv) of volatile organic compounds.

Compounds	K×10000 [a]	OT1 [b]	OT2 [c]	OT3 [d]	OT4 [e]	OT5 [f]	OT6 [g]	OT [h]
Methanol			24745255		100000			100000
Acetaldehyde			825		210			210
Formic acid				27925				27925
Ethanol			273766		10000			10000
2-Propenal			1564		210			210
Propanal			419					419
Acetone			17618249		100000			100000
Acetic acid			2435	162	1000	562		162
Dimethyl sulfide			1179		1			1
Butan-2-one	48	59624	149168		10000			10000
Propanoic acid			1881	33				33
Methyl acetate			561124					561124
Dimethyl sulfoxide			940		1			1
Ethyl acetate	53	1269	241973					1269
Butanoic acid			53	4	1			1
trans-2-Pentenal						437		437
Pentanal			117			11		11
Ethyl propionate			1682					1682
3-Methylbutanoic acid			19					19
Pentanoic acid			9					9
Cyclohexane			727665					727665
trans-2-Hexenal							1	1
Hexanal	6	11	69			12	0.27	0.27
4-Methylpentan-2-one					470			470
Butyl acetate	5	29	3385					29
Ethyl butyrate			8					8
Ethyl isobutyrate			5					5
Hexanoic acid			127			635		127

Compounds	K×10000 [a]	OT1 [b]	OT2 [c]	OT3 [d]	OT4 [e]	OT5 [f]	OT6 [g]	OT [h]
Toluene			87827		2140-4680			2140
Ethenyl benzene			8250		47-100			47
Ethyl benzene			39323					39323
2,4-Heptadienal						8		8
trans-2-Heptenal						19		19
Heptanal	3	30	39			10	0.19	0.19
Heptan-2-one	5	30	1463				1	1
1,2,4-Trimethylbenzene			24545					24545
trans-2-Octenal						4	1	1
3-Octen-2-one							1	1
Octanal	2	11	2			2	0.08	0.08
1-Octen-3-ol							1	1
Octan-2-one	3	27						27
Propyl butanoate			2078					2078
α-Pinene			3251					3251
β-Pinene			5961					5961
Limonene			6864			130		130
trans,trans-2,4-Nonadienal							0.04	0.04
tran-2-Nonenal							0.02	0.02
Nonanal			59			4	0.45	0.45
Nonan-2-one							5	5
Hexyl acetate			307					307
trans, trans-2,4-Decadienal							0.37	0.37
trans-2-Decenal						1	0.43	0.43
Decanal			63			1	0.41	0.41

[a] van Ruth, et al. [1]; [b] Morales, et al. [2]; [c] Nagata and Takeuchi [3]; [d] Nielsen, et al. [4]; [e] Leonardos, et al. [5]; [f] Xu, et al. [6]; [g] Yang, et al. [7]; [h] The lowest OT selected from publications was used in this study.

References:

1. van Ruth, S. M.; Grossmann, I.; Geary, M.; Delahunty, C. M., Interactions between artificial saliva and 20 aroma compounds in water and oil model systems. *J. Agr. Food Chem.* **2001,** 49, (5), 2409-2413.
2. Morales, M. T.; Luna, G.; Aparicio, R., Comparative study of virgin olive oil sensory defects. *Food Chem.* **2005,** 91, (2), 293-301.
3. Nagata, Y.; Takeuchi, N., Measurement of odor threshold by triangle odor bag method. *Odor measurement review* **2003,** 118, 118-127.
4. Nielsen, G. D.; Hansen, L. F.; Andersen, B.; Poulsen, N.; Melchior, O., Indoor air guideline levels for formic, acetic, propionic and butyric acid. *Indoor Air* **1998,** 8, (S5), 8-24.
5. Leonardos, G.; Kendall, D.; Barnard, N., Odor threshold determination of 53 odorant chemicals. *J. Air Pollut. Control Assoc.* **1969,** 19, (2), 91-95.

6. Xu, L.; Yu, X.; Li, M.; Chen, J.; Wang, X., Monitoring oxidative stability and changes in key volatile compounds in edible oils during ambient storage through HS-SPME/GC–MS. *Int. J. Food Prop.* **2017,** 20, (sup3), S2926-S2938.
7. Yang, D. S.; Shewfelt, R. L.; Lee, K.-S.; Kays, S. J., Comparison of odor-active compounds from six distinctly different rice flavor types. *J. Agr. Food Chem.* **2008,** 56, (8), 2780-2787.
